# Metabolic Interactions between *Brachypodium* and Pseudomonas fluorescens under Controlled Iron-Limited Conditions

**DOI:** 10.1128/mSystems.00580-20

**Published:** 2021-01-05

**Authors:** Rene M. Boiteau, Lye Meng Markillie, David W. Hoyt, Dehong Hu, Rosalie K. Chu, Hugh D. Mitchell, Ljiljana Pasa-Tolic, Janet K. Jansson, Christer Jansson

**Affiliations:** a College of Earth, Ocean, and Atmospheric Sciences, Oregon State University, Corvallis, Oregon, USA; b Environmental Molecular Sciences Laboratory, Pacific Northwest National Laboratory, Richland, Washington, USA; c Biological Sciences Division, Pacific Northwest National Laboratory, Richland, Washington, USA; University of Dundee

**Keywords:** iron, grass, siderophore

## Abstract

Iron (Fe) availability has well-known effects on plant and microbial metabolism, but its effects on interspecies interactions are poorly understood. The purpose of this study was to investigate metabolite exchange between the grass *Brachypodium distachyon* strain Bd21 and the soil bacterium Pseudomonas fluorescens SBW25::gfp/lux (SBW25) during Fe limitation under axenic conditions. We compared the transcriptional profiles and root exudate metabolites of *B. distachyon* plants grown semihydroponically with and without SBW25 inoculation and Fe amendment. Liquid chromatography-mass spectrometry analysis of the hydroponic solution revealed an increase in the abundance of the phytosiderophores mugineic acid and deoxymugineic acid under Fe-limited conditions compared to Fe-replete conditions, indicating greater secretion by roots presumably to facilitate Fe uptake. In SBW25-inoculated roots, expression of genes encoding phytosiderophore biosynthesis and uptake proteins increased compared to that in sterile roots, but external phytosiderophore abundances decreased. P. fluorescens siderophores were not detected in treatments without Fe. Rather, expression of SBW25 genes encoding a porin, a transporter, and a monooxygenase was significantly upregulated in response to Fe deprivation. Collectively, these results suggest that SBW25 consumed root-exuded phytosiderophores in response to Fe deficiency, and we propose target genes that may be involved. SBW25 also altered the expression of root genes encoding defense-related enzymes and regulators, including thionin and cyanogenic glycoside production, chitinase, and peroxidase activity, and transcription factors. Our findings provide insights into the molecular bases for the stress response and metabolite exchange of interacting plants and bacteria under Fe-deficient conditions.

**IMPORTANCE** Rhizosphere bacteria influence the growth of their host plant by consuming and producing metabolites, nutrients, and antibiotic compounds within the root system that affect plant metabolism. Under Fe-limited growth conditions, different plant and microbial species have distinct Fe acquisition strategies, often involving the secretion of strong Fe-binding chelators that scavenge Fe and facilitate uptake. Here, we studied interactions between P. fluorescens SBW25, a plant-colonizing bacterium that produces siderophores with antifungal properties, and *B. distachyon*, a genetic model for cereal grain and biofuel grasses. Under controlled growth conditions, bacterial siderophore production was inhibited in the root system of Fe-deficient plants, bacterial inoculation altered transcription of genes involved in defense and stress response in the roots of *B. distachyon*, and SBW25 degraded phytosiderophores secreted by the host plant. These findings provide mechanistic insight into interactions that may play a role in rhizosphere dynamics and plant health in soils with low Fe solubility.

## Abstract (precis)

Rhizosphere bacteria influence the growth of their host plant by consuming and producing metabolites, nutrients, and antibiotic compounds within the root system that affect plant metabolism. Under Fe-limited growth conditions, different plant and microbial species have distinct Fe acquisition strategies, often involving the secretion of strong Fe-binding chelators that scavenge Fe and facilitate uptake.

## INTRODUCTION

Root-associated microbes impact the health of their host through the consumption and production of molecules that act as chemical cues and reservoirs of nutrients and energy. These metabolic interactions are affected by environmental stressors. Iron (Fe) deficiency is particularly problematic for crop production in calcareous and alkaline soils, where the solubility of inorganic Fe species is below the 10^−6^ to 10^−5^ M required for optimal plant growth rates (1–4). Iron is required for photosynthesis and energy transfer in plants but also for the metabolism of rhizosphere microbes (5, 6). In addition to directly limiting plant growth, Fe deficiency can alter the composition of exudates that transfer energy, facilitate Fe or nutrient uptake, or act as signaling molecules between the host plant and root-associated bacteria. While changes in plant Fe nutrition status have large effects on the rhizosphere microbiome, the underlying molecular mechanisms are poorly understood (53).

Many soil microbes and plants respond to Fe deficiency by secreting siderophores (referred to as phytosiderophores in plants), chelating agents that solubilize Fe in a form that can be taken up by specific membrane transporters (7, 8). Soil bacteria often possess multiple siderophore biosynthesis and uptake pathways that are utilized under different growth conditions. Among the common taxa found in soils, *Pseudomonas* spp. are particularly adept at producing a wide range of siderophores, including pyoverdine, pyochelin, and ferrocin (8). Secretion of these compounds alters metal homeostasis within the rhizosphere and can improve plant growth by suppressing pathogenic species (9). Many pseudomonads also possess transporters for exogenous siderophores that they do not produce (10, 11). When utilizing exogenous siderophores, these pseudomonads no longer produce their own siderophores (12), thus shifting the energetic and nutrient cost of production to other organisms (13).

While microbial utilization of exogenous bacterial siderophores is well studied, less is known about siderophore interactions between plants and rhizosphere bacteria (14, 15). In some cases, siderophores produced by rhizosphere bacteria may contribute to plant Fe nutrition. Amendment of bacterial siderophores, such as pyoverdine and ferrioxamine, have been found to promote Fe uptake by some plants (16–18). It remains unclear whether this effect is from direct assimilation of microbial siderophores by plants, ligand exchange, or reduction-based Fe uptake. Grasses also respond to Fe deficiency by secreting phytosiderophores from root tips that facilitate Fe solubilization and uptake (4, 14, 19). Phytosiderophores are rapidly degraded in soils by some microorganisms (20–22). Previous studies have found that some Gram-negative bacteria can assimilate carbon from phytosiderophores based on lipid biomarker analysis after incubation of ^13^C-labeled phytosiderophores in grassland soils (23). The specific taxa capable of phytosiderophore metabolism and the genes involved are currently unknown.

The goal of this study was to assess the biochemical pathways by which plants and plant-associated microorganisms interact and acquire Fe under Fe-replete and -deficient growth conditions. Experiments were performed in plant-microbe cocultures of Brachypodium distachyon, a genomics model for cereal grain species and bioenergy grasses (24), and Pseudomonas fluorescens SBW25::gfp/lux (SBW25), a plant-colonizing bacterium. We conducted controlled growth experiments with sterile and SBW25-inoculated plants in a semihydroponic growth system. Our experimental design enabled us to analyze the transcriptional response of the plants and bacteria under different growth conditions as well as to determine changes in the metabolite composition of the growth medium. We found that genes involved in phytosiderophore synthesis and uptake were upregulated in *B. distachyon* roots inoculated with SBW25 versus sterile controls, likely due to the consumption of phytosiderophores by SBW25. Several transport-related genes were upregulated by the root-associated SBW25 bacteria in response to Fe deprivation, which identifies candidate genes potentially involved in the uptake of phytosiderophore. These findings illuminate the metabolic mechanisms by which root-associated bacteria may affect graminaceous host health under Fe-deficient conditions.

## RESULTS

### Effects of Fe and bacteria on plant growth.

Growth experiments were carried out in sterile, semihydroponic containers with a defined nutrient composition. Silica beads were added to represent a soil matrix (25, 26). By comparison to “natural” soil, this artificial soil matrix allowed us to (i) minimize variation between replicates, (ii) reproducibly separate the rhizosphere matrix from the roots for biomass weight and transcriptional analysis, and (iii) easily recover the nutrient solutions containing root exudates for metabolomic analysis.

**FIG 1:**
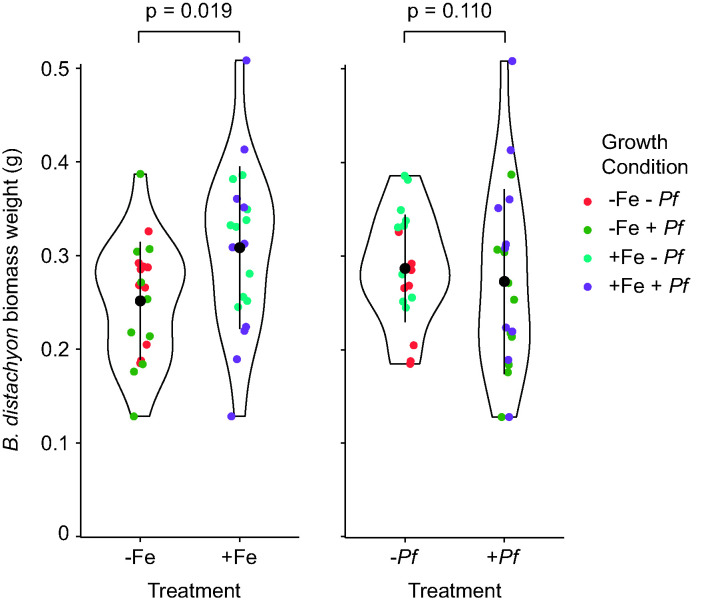
Violin plots of *B. distachyon* biomass (wet weight) after 28 days of growth comparing the four experimental growth conditions, sterile plants with Fe (+Fe − *Pf*), and without Fe (−Fe − *Pf*), and P. fluorescens SBW25-inoculated plants with Fe (+Fe + *Pf*) and without Fe (−Fe + *Pf*). Vertical lines indicate ±1 standard deviation, and black points indicate means. Statistical significance (*P* value; Wilcoxon rank sum test) of differences between groups is shown above the plots. Measurements of individual plants are shown as colored points.

**FIG 2:**
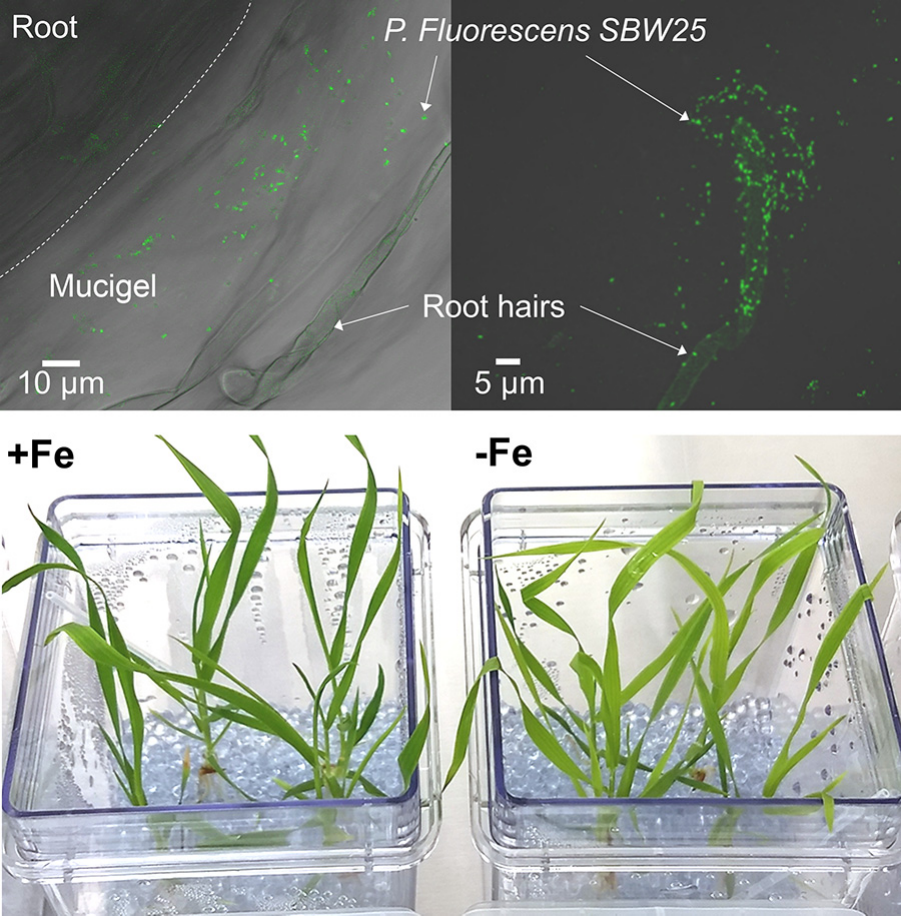
(Top) Confocal microscopy images showing roots with a patch of GFP-tagged P. fluorescens SBW25 within mucigel surrounding the roots and root hairs. The dashed line indicates the border of the root and mucigel. (Bottom) *B. distachyon* plants after 28 days of growth with and without added Fe.

We found that growth in Hoagland medium without Fe (−Fe) compared to growth in medium with Fe (+Fe) resulted in a significant decrease in *B. distachyon* biomass based on a Wilcoxon rank sum test (*P* value = 0.018) (Fig. 1). Furthermore, chlorosis was observed in the −Fe treatment, as evidenced by yellowing of the leaves (Fig. 2). Bacterial inoculation had no significant effect on *B. distachyon* biomass (*P* value = 0.11) (Fig. 1).

Previous field-based studies have demonstrated that SBW25 effectively colonizes both the root and root-associated soils of grasses (27). Confocal microscopy was used to observe the colonization patterns of green fluorescent protein (GFP)-tagged SBW25 on the roots of *B. distachyon* grown semihydroponically. While some bacteria were found directly on the root tip, most bacteria colonized zones distal from the tip, particularly in regions where mucigel adhered the silica substrate to the root (Fig. 2). Other bacteria were motile within the nutrient solution. Similar colonization patterns along roots of wheat and barley by P. fluorescens have been observed in natural soils (28, 29). Our controlled experimental design thus has features that are relevant to understanding the rhizosphere behavior of *Pseudomonas* spp. in natural soil environments.

### Exuded metabolites are altered by Fe deprivation and SBW25 inoculation.

**FIG 3:**
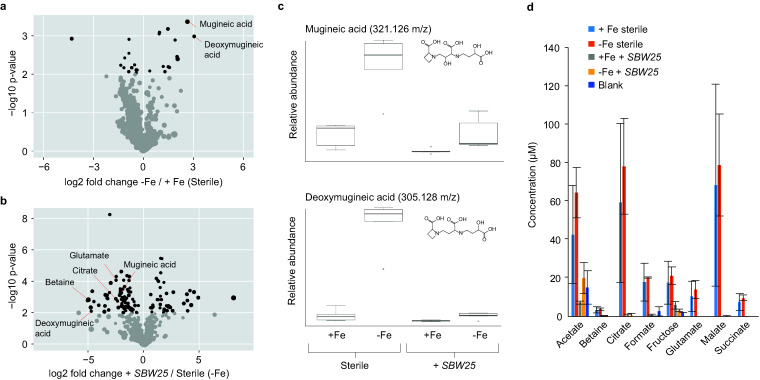
Volcano plot of LC-MS-detected metabolites showing statistical significance (*n* = 5) versus magnitude of change in abundance between (a) −Fe and +Fe treatments under sterile conditions and (b) P. fluorescens SBW25 and sterile treatments under −Fe conditions. Features with *P* values of <0.01 are shown in black. (c) Box plot of relative abundances of mugineic acid and deoxymugineic acid measured by LC-MS across the four experimental conditions. (d) Abundance of major metabolites quantified by NMR. Error bars represent 1 standard deviation (*n* = 5).

The metabolomic differences between Fe-replete (+Fe) and Fe-limited (−Fe) conditions revealed specific metabolites that were produced and/or imported by the plants and bacteria. Overall, 1,059 mass spectrometry features were detected by liquid chromatography-mass spectrometry (LC-MS). Fe deprivation resulted in the differential detection (*P* < 0.01) of 27 of these features in sterile plants and 20 features in plants with SBW25 (Fig. 3a), compared to +Fe conditions. These features were searched against the BioCyc (https://biocyc.org) and PlantCyc (https://plantcyc.org/) metabolic pathway databases to generate putative annotations based on matches to known molecules within predicted metabolic pathways of *B. distachyon* and P. fluorescens SBW25. The greatest change was observed for features corresponding to protonated forms of mugineic acid (321.127 *m/z*) and deoxymugineic acid (305.132 *m/z*) (Fig. 3c). These features were 6- to 8-fold more abundant in −Fe treatments than +Fe treatments in samples without SBW25 and 2- to 3-fold more abundant in samples with SBW25.

Inoculation with SBW25 resulted in the differential detection of 114 and 109 features by LC-MS (*P* value < 0.01) in Fe-replete and -deficient conditions, respectively (Fig. 3b). Of these, 31 features were less abundant when SBW25 was present in both +Fe and −Fe conditions. These included mugineic acid and deoxymugineic acid (Fig. 3c) as well as other metabolites previously observed in *B. distachyon* root exudates, including betaine (118.085 *m/z*), citrate (215.014 *m/z*), malate (135.029 *m/z*), and glutamate (148.0579 *m/z*) (25). Twenty-five metabolites were >3-fold more abundant in the presence of SBW25. These correspond to compounds that were produced by the bacteria or released by *B. distachyon* in response to SBW25. The feature with the largest fold increase (479.183 *m/z*) matched the protonated mass of *N*-acetyl-β-d-glucosamine-1,6-anhydro-*N*-acetyl-β-d-muramate (C_19_H_29_N_2_O_12_), a predicted SBW25 metabolite involved in peptidoglycan construction and recycling (30).

Nuclear magnetic resonance (NMR) metabolomic analysis provided absolute quantitation of abundant species (31). The major metabolites identified (Fig. 3) corresponded to osmolytes (betaine), anions of organic acids (malate, glutamate, formate, acetate, and citrate), and sugars (fructose), showing good agreement with the LC-MS results, plus some additional compounds. Iron deprivation did not have a significant effect on the total concentration of these major metabolites (*P* > 0.1, Wilcoxon rank sum test). Their concentration decreased by over 90%, however, in treatments with SBW25 inoculation (*P* < 0.01, Wilcoxon rank sum test).

### Upregulated phytosiderophore gene transcription in response to SBW25.

**FIG 4:**
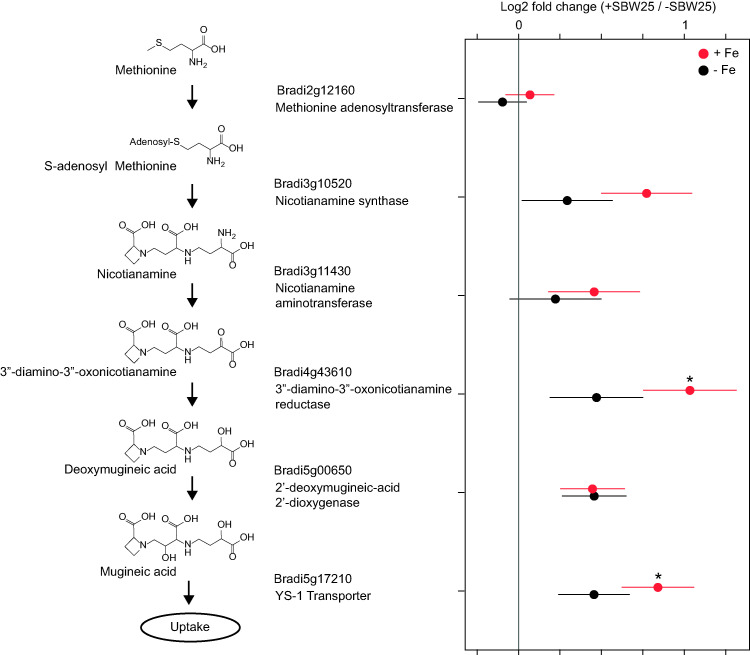
Comparison of transcript abundance in +SBW25- versus −SBW25-treated roots of *B. distachyon* grown with and without Fe amendment, showing the response of the most highly expressed copy of genes for the biosynthesis of phytosiderophores (left). Error bars represent a 1-log 2-fold change standard error (*n* = 5). Comparisons with adjusted *P* values of <0.05 are marked with asterisks.

To determine how phytosiderophore production differed between growth conditions, we investigated the transcriptional response of *B. distachyon* roots. Phytosiderophore biosynthesis is carried out by five enzymatic steps (32). The first involves a methionine adenosyltransferase that activates methionine with ATP. The second enzyme, nicotianamine synthase, combines three molecules of *S*-adenosyl-l-methionine to create nicotianamine. The third step involves a nicotianamine aminotransferase that catalyzes the transamination reaction between nicotianamine and 2-oxoglutarate, which is the first step in mugineic acid biosynthesis unique to graminaceous plants (33). Fourth, a 3″-deamino-3″-oxonicotianamine reductase reduces the 3″-oxo group to an alpha-hydroxy group to make deoxymugineic acid. Finally, this product can be hydroxylated to produce mugineic acid by a 2′-deoxymugineic-acid 2′-dioxygenase. Mugineic acid and deoxymugineic acid are secreted by roots and then reassimilated via transporters that are family members of the Yellow Stripe 1 (YS1) group of proteins. *B. distachyon* possesses 19 different homologues of YS1 (34). Some of these are involved in the uptake of metal-phytosiderophore complexes, while others have roles for internal metal translocation in plants. Figure 4 shows the regulation of the most abundantly expressed copy of each gene in the roots (based on transcript counts) and highlights the genes that are most likely of primary importance in phytosiderophore production in roots. The most highly expressed YS1-like transporter observed in our study (Bradi5g17210) matches the transporter implicated previously as the major controller of Fe-phytosiderophore uptake from soils (34). Comparison of gene transcripts showed greater expression in SBW25-inoculated than sterile plants of five of the phytosiderophore biosynthesis and uptake genes found to be most transcriptionally abundant in the roots under both −Fe and +Fe conditions (Fig. 4), indicating that phytosiderophore production likely increased in the presence of SBW25.

### Altered plant metabolism, defense, transport, and regulation by SBW25 inoculation.

The transcriptional abundances of 852 and 526 genes were significantly different (adjusted *P* value < 0.05) in plants inoculated with SBW25 compared to sterile plants in the +Fe and −Fe treatments, respectively. Genes that were differentially expressed by more than a factor of 2 were characterized based on gene ontology (GO) terms obtained from PhytoMine (https://phytozome.jgi.doe.gov/phytomine/) and predicted involvement in biosynthetic pathways obtained from the PlantCyc plant metabolic pathway database (Table 1; also, see Table S1 in the supplemental material).

**TABLE 1:** Transcriptional response of *B. distachyon* roots to SBW25 under +Fe and −Fe growth conditions^*a*^

Gene ID	+Fe		−Fe		Annotation^*b*^
	Log_2_ fold change	*P*_adj_	Log_2_ fold change	*P*_adj_	
Biosynthesis and metabolism					
Bradi2g11870	−1.12 ± 0.28	7.4E−03	−0.89 ± 0.28	4.9E−02	Glycosyl hydrolase
Bradi1g00950	−1.06 ± 0.26	5.0E−03	−0.58 ± 0.25	1.4E−01	2-Oxoglutarate oxygenase
Bradi2g49067	1.01 ± 0.28	1.5E−02	0.72 ± 0.28	9.5E−02	UDP-glucosyl transferase
Bradi1g22340	1.01 ± 0.28	1.5E−02	0.13 ± 0.27	8.1E−01	Cytochrome P450
Bradi4g31960	1.03 ± 0.21	6.1E−04	0.53 ± 0.21	1.1E−01	Pyridoxal phosphate-dependent transferase
Bradi4g43610	1.04 ± 0.28	1.4E−02	0.47 ± 0.28	2.9E−01	2′-Deoxymugineic acid 2′-dioxygenase
Bradi3g13700	1.05 ± 0.24	2.1E−03	0.56 ± 0.23	1.2E−01	d-Arabinono-1,4-lactone oxidase
Bradi3g18160	1.05 ± 0.28	1.4E−02	0.77 ± 0.28	8.1E−02	d-Arabinono-1,4-lactone oxidase
Bradi4g08830	1.05 ± 0.27	8.9E−03	0.87 ± 0.27	4.4E−02	Aldolase-type TIM barrel
Bradi3g06330	1.05 ± 0.26	6.5E−03	1.14 ± 0.26	4.1E−03	Cytochrome P450
Bradi1g59570	1.05 ± 0.28	1.2E−02	−0.57 ± 0.28	1.8E−01	Gibberellin 2-oxidase
Bradi4g43380	1.06 ± 0.22	4.1E−04	0.55 ± 0.21	1.0E−01	Cytochrome P450
Bradi1g06730	1.12 ± 0.27	5.1E−03	0.81 ± 0.27	6.1E−02	Cytochrome P450
Bradi2g44150	1.14 ± 0.24	7.0E−04	0.68 ± 0.24	7.2E−02	Cytochrome P450
Bradi2g44160	1.28 ± 0.27	6.8E−04	0.89 ± 0.27	4.0E−02	Cytochrome P450
Bradi1g64120	1.32 ± 0.24	2.9E−05	−0.10 ± 0.23	8.2E−01	Galactinol synthase

Stress and defense					
Bradi1g57280	−1.26 ± 0.28	2.1E−03	−1.24 ± 0.28	4.4E−03	Plant thionin
Bradi1g57285	−1.17 ± 0.25	7.1E−04	−1.17 ± 0.24	1.5E−03	Plant thionin
Bradi4g14920	0.85 ± 0.27	3.9E−02	1.01 ± 0.27	1.8E−02	Chitinase
Bradi5g27170	−1.36 ± 0.27	2.6E−04	−0.82 ± 0.26	5.1E−02	Peroxidase
Bradi2g20850	−1.35 ± 0.27	2.8E−04	−1.05 ± 0.27	1.2E−02	Peroxidase
Bradi1g38310	−1.12 ± 0.28	6.1E−03	−0.80 ± 0.27	6.6E−02	Peroxidase
Bradi4g24650	−0.57 ± 0.23	1.2E−01	−1.06 ± 0.23	2.2E−03	ABA/WDS induced protein
Bradi2g37060	0.21 ± 0.26	6.7E−01	1.32 ± 0.26	6.6E−04	Peroxidase
Bradi3g55850	1.18 ± 0.27	2.8E−03	0.42 ± 0.27	3.1E−01	Peroxidase
Bradi4g25660	1.20 ± 0.28	4.5E−03	0.60 ± 0.28	1.7E−01	Peroxidase

Transport					
Bradi5g14400	0.57 ± 0.20	6.7E−02	1.00 ± 0.20	6.8E−04	Oligopeptide transport related
Bradi5g14410	0.65 ± 0.25	9.7E−02	1.03 ± 0.25	7.8E−03	Oligopeptide transmembrane transporter
Bradi3g35390	1.01 ± 0.28	1.9E−02	0.57 ± 0.28	2.0E−01	ATPase activity

Regulation					
Bradi1g56841	0.45 ± 0.25	2.6E−01	1.11 ± 0.24	2.3E−03	FAR1 domain-containing protein
Bradi1g58057	1.00 ± 0.24	5.4E−03	1.04 ± 0.24	4.7E−03	NAC domain-containing protein
Bradi3g22040	1.07 ± 0.23	1.0E−03	0.17 ± 0.23	6.6E−01	bZIP transcription factor
Bradi2g40582	1.28 ± 0.25	2.8E−04	0.20 ± 0.25	6.5E−01	bZIP transcription factor
Bradi3g07540	1.74 ± 0.27	3.4E−07	−0.27 ± 0.26	5.3E−01	bZIP transcription factor

a Fold change values were calculated as (value with SBW25)/(value without SBW25). Values are means ± standard errors. *P*_adj_, adjusted *P* value.

b GO terms and pathways are included in Table S1.

10.1128/mSystems.00580-20.1 TABLE S1 Transcriptional response and adjusted *P* value (Padj) of *B. distachyon* roots to SBW25 under +Fe and −Fe growth conditions, including GO terms and pathways. Download Table S1, PDF file, 0.1 MB. Copyright © 2021 Boiteau et al. 2021 Boiteau et al. This content is distributed under the terms of the Creative Commons Attribution 4.0 International license.

Numerous differentially expressed genes encoded oxidoreductase proteins (GO:0016705) likely involved in metabolite, hormone, and defense protein synthesis. In addition to the 2′-deoxymugineic-acid 2′-dioxygenase (Bradi4g43610), a putative pyridoxal phosphate-dependent transferase involved in the l-methionine salvage cycle (Bradi4g31960), was upregulated in SBW25-inoculated roots, consistent with increased production of phytosiderophore precursors. Six genes that were upregulated in SBW25-inoculated roots were annotated as heme-containing cytochrome P450 enzymes. Of these, two genes were attributed to biosynthesis pathways in PlantCyc. Bradi1g22340 encodes an enzyme that is predicted to convert (4-hydroxyphenyl)acetonitrile to (*S*)-4-hydroxymandelonitrile as part of the biosynthesis of dhurrin, a cyanogenic glucoside (35). Bradi4g43610 encodes a protein that is predicted to inactivate the defense response phytohormone jasmonyl-l-isoleucine (36, 37). These results suggest that *B. distachyon* modulated antibiotic and phytohormone synthesis in response to colonization. Fold changes were generally lower under −Fe growth conditions, possibly to conserve a limited Fe inventory. Three of the differentially expressed genes encode defense-related proteins (GO:0006952, GO:0042742, and GO:0050832), including thionins (Bradi1g57285 and Bradi1g57280) that were downregulated by inoculation with SBW25 and an enzyme with chitinase activity (Bradi4g14920) that was upregulated by SBW25. Similar responses were observed under −Fe and +Fe conditions. These results suggest that *B. distachyon* altered root defense mechanisms in response to SBW25 colonization.

Differentially expressed genes involved in root response to oxidative stress (GO:0006979) included six genes encoding peroxidases (Table 1). Expression of three peroxidase genes within this group was repressed by SBW25 (Bradi5g27170, Bradi2g20850, and Bradi1g38310), while expression of three other peroxidase genes was induced by SBW25 (Bradi2g37060, Bradi3g55850, and Bradi4g25660), suggesting that bacterial colonization affected oxidative stress management in the roots.

Three transport-associated genes (GO:0006810) were upregulated in SBW25-inoculated roots. Bradi5g14400 encodes a putative membrane-associated transport-associated protein, Bradi5g14410 encodes a putative proton-dependent oligopeptide active membrane transporter, and Bradi3g35390 was annotated as an ATP-binding cassette transporter. These proteins appear to be components of one or more systems for active transport of small molecules that were synthesized or taken up in response to SBW25 colonization.

Five root genes involved in the regulation of transcription (GO:0006355) were upregulated in SBW25-inoculated roots, indicating specific pathways by which gene expression responses may be controlled. Three of these genes (Bradi3g22040, Bradi2g40582, and Bradi3g07540) encode basic leucine zipper transcription factors, a class of regulators that commonly play a role in biotic and abiotic stress responses under +Fe conditions, although a weaker response was observed under −Fe conditions. Two other regulatory genes encode FAR1 (far-red-impared response) and NAC (no apical meristem/*Arabidopsis thaliana* activating factor/cup-shaped cotyledon) DNA binding domain-containing proteins (Bradi1g56841 and Bradi1g58057).

### Induction of transport-associated bacterial genes by Fe starvation.

The relative abundances of transcripts from SBW25 were compared between −Fe and +Fe treatments. Four genes were significantly upregulated (adjusted *P* value < 0.05) under Fe deprivation (Table 2). The presence of Pfam domains was computed by BioCyc (38). The most significantly upregulated gene was PFLU_RS26950, encoding the outer membrane protein W porin (Pfam-PF03922). This receptor is the target by which the antimicrobial agent colicin S4 enters Escherichia coli but otherwise has no known substrate (39). Additional upregulated genes included those encoding a transport-associated oligonucleotide/oligosaccharide binding (OB) domain protein (PFLU_RS13625, Pfam-PF03459) within the same family that binds metal cofactors such as molybdate and molybdopterins (40), an antibiotic biosynthesis monooxygenase (PFLU_RS24305, Pfam-PF03992), and a hypothetical protein with no annotation (PFLU_RS02795). These putative annotations are consistent with functions associated with the transport and processing of metal-organic complexes. SBW25 is known to synthesize only one siderophore (pyoverdine), with 31 genes involved in biosynthesis, transport, and regulation (41). Low copy numbers of the pyoverdine biosynthesis and uptake transcripts (genes PFLU_RS12435 to PFLU_RS12485) were detected, and no significant difference was observed between +Fe and −Fe treatments (Table S2).

**TABLE 2:** Transcriptional response of root-associated P. fluorescens SBW25 significantly affected by Fe deprivation

Gene ID	Log_2_ fold change^*a*^	*P*_adj_	Feature class	Annotation
PFLU_RS26950	1.66 ± 0.27	5.0 E−6	Pfam-PF03922	Outer membrane protein
PFLU_RS13625	1.58 ± 0.33	2.1 E−3	Pfam-PF03459	Transmembrane transporter
PFLU_RS02795	1.50 ± 0.30	2.1 E−3	None	Hypothetical protein
PFLU_RS24305	1.45 ± 0.30	2.1 E−3	Pfam-PF03992	Monooxygenase

a Fold change values were calculated as (−Fe value)/(+Fe value). Values are means ± standard errors.

10.1128/mSystems.00580-20.2 TABLE S2 Transcriptional response of P. fluorescens SBW25 to Fe deprivation: genes with predicted pyoverdine biosynthesis functions. Download Table S2, PDF file, 0.1 MB. Copyright © 2021 Boiteau et al. 2021 Boiteau et al. This content is distributed under the terms of the Creative Commons Attribution 4.0 International license.

## DISCUSSION

The results from this controlled growth experiment depict the Fe stress response of interactions between *B. distachyon* and P. fluorescens SBW25. First, we evaluated the effect of Fe deprivation on *B. distachyon*. Observations of reduced biomass under −Fe conditions (Fig. 1) demonstrated Fe limitation of *B. distachyon* growth. Our metabolomic results confirmed increased exudation of the *B. distachyon* phytosiderophores mugineic acid and deoxymugineic acid under −Fe conditions. While overall exudation was slightly greater in the −Fe than the +Fe treatments, the increase in phytosiderophore abundance was much greater than that of any other metabolites detected (Fig. 3). These outcomes are consistent with well-established grass responses to Fe deficiency (34) and provide a baseline for comparison to the SBW25-inoculated treatments.

Comparisons of transcriptomic and metabolomic results with and without SBW25 suggest a bacterial role in phytosiderophore degradation. Although phytosiderophore biosynthesis gene transcripts were more abundant in SBW25-inoculated roots than in sterile roots (Fig. 4), phytosiderophore abundances measured by LC-MS were lower in the treatments with SBW25 (Fig. 3). Together, these results indicate that (i) *B. distachyon* increased phytosiderophore production in response to bacterial colonization and (ii) SBW25 consumed or otherwise degraded these phytosiderophores, resulting in overall lower abundances within the root exudate solution. These results differ from previous observations by Walter et al. showing that Pseudomonas putida (*P. putida*) strains did not degrade phytosiderophores from maize and barley in culture (42), suggesting that not all pseudomonads have this ability. Additional experiments with purified phytosiderophores added to axenic cultures in the absence of the plant would help to demonstrate assimilation of phytosiderophores by P. fluorescens SBW25.

We next evaluated the response of SBW25 to Fe deprivation in the presence of *B. distachyon*. It is well established that Fe-deficient *Pseudomonas* spp. secrete pyoverdine in pure culture (43, 44) and in the rhizosphere of plants (15, 45). In this study, however, the genes involved in pyoverdine production were not upregulated in the −Fe conditions. Furthermore, SBW25 pyoverdine concentrations in the exudate solutions were below detection limits (20 nM). One explanation is that SBW25 possesses a molecular mechanism to utilize mugineic acid and deoxymugineic acid secreted by *B. distachyon* roots as a source of Fe rather than expend energy on endogenous siderophore production. Pyoverdine production is regulated by several environmental signals and regulatory pathways. Expression is strongly repressed by the presence of Fe (46), and it is possible that SBW25 in the −Fe medium treatments satisfied its Fe quota by uptake of Fe-bound mugineic acid, deoxymugineic acid, or some other plant-derived chelate that solubilized residual Fe from the roots or the silica bead substrate. Pyoverdine synthesis is also regulated by a number of quorum-sensing two-component systems and second messengers (46). In particular, pyoverdine gene expression is modulated by the Gac/Rsm system, which also controls the switch from a planktonic to a biofilm lifestyle (47). Our sampling of SBW25 and metabolites within the nutrient solution may select for planktonic cells that have inhibited pyoverdine production compared to biofilm-attached cells. Alternatively, mugineic acid or some other plant-derived molecule may signal SBW25 to downregulate pyoverdine production.

The degradation of phytosiderophores by SBW25 implies that this strain possesses transporters and enzymes for taking up and metabolizing mugineic acid and deoxymugineic acid. P. fluorescens regulates the transcription of several exogenous siderophore transporters through cell surface signaling mechanisms that are induced by binding of the siderophore to a membrane receptor, so that gene expression reflects external siderophore abundance (48). Since mugineic acid and deoxymugineic acid were over 2-fold more abundant in the nutrient solution of −Fe treatments than that of +Fe treatments, we investigated the transcriptional response of SBW25 to Fe deprivation in order to identify gene targets that potentially encode transporters or enzymes involved in phytosiderophore utilization. We identified three genes encoding a transporter (PFLU_RS13625), an outer membrane porin (PFLU_RS26950), and an oxidoreductase (PFLU_RS13625) that were upregulated by Fe deprivation. These genes may be involved in phytosiderophore uptake and decomposition. Further work is needed to validate these proposed roles by generating strains with these genes knocked out and determine whether phytosiderophore uptake is hindered. While our results do not rule out other possible roles for the upregulated genes or the involvement of other genes in phytosiderophore uptake, they are consistent with a mechanism for direct utilization of phytosiderophores as an Fe source by SBW25.

Previous studies have reached conflicting conclusions regarding mechanisms of *Pseudomonas* spp. Fe uptake in root systems. Jurkevitch et al. found that P. putida can assimilate radiolabeled Fe bound to mugineic acid (49). They observed diminished short-term uptake by a siderophore-deficient mutant and attributed Fe uptake to ligand exchange between bacterial siderophores and phytosiderophores. However, a subsequent study by Marschner et al. observed that P. fluorescens decreased production of pyoverdine, determined by ice nucleation reporter activity, in the roots of phytosiderophore-producing grasses compared to grasses treated with foliar Fe to alleviate plant Fe stress and phytosiderophore release (50). These authors suggested that phytosiderophore-bound Fe may be directly assimilated by P. fluorescens, reducing the need for pyoverdine production. Although indirect uptake of phytosiderophore-bound Fe is possible through ligand exchange with pyoverdine, this mechanism still requires pyoverdine production to remove Fe from phytosiderophores. Our study provides additional evidence that root-associated P. fluorescens degrades phytosiderophores under Fe deprivation without producing pyoverdine and uncovers candidate transporters potentially involved in phytosiderophore uptake. Only one other study has described bacterial isolates capable of phytosiderophore decomposition (51). These strains were isolated from the roots of Fe-deficient barley, and one was judged to belong to *Pseudomonas* spp., although no genetic information was available. Our findings build upon this prior work by providing fundamental insight into the molecular mechanism and possible genetic basis of phytosiderophore acquisition by a soil bacterium.

The semihydroponic experimental design used in this study has several important limitations and distinctions from natural soils where Fe limitation is expected. In soils, Fe availability generally correlates with pH (14), and thus siderophore production predominantly occurs in environments where soil pH is neutral to alkaline (45), whereas the nutrient solution used in this study was slightly acidic (pH 5.8). Furthermore, in the semihydroponic system, the root zone was always saturated with water and the growth medium was significantly simplified compared to natural soils. In natural rhizosphere soils, siderophore production could occur under environmental pH, moisture, and minerology regimens that differ from those in this study. Diffusion rates are also higher in the semihydroponic system, and this may reduce or eliminate spatial gradients in root colonization or phytosiderophores that are normally concentrated behind the root tip where they are released. Pyoverdine may still play a key role in microbial Fe acquisition, particularly at sites of lateral root emergence along mature axes. Finally, phytosiderophores are released in diurnal pulses with maximum exudation rates after the onset of day light (34). Here, samples were analyzed during the period when maximum secretion was expected, but root-associated microbes may rely on their own siderophores to mediate Fe uptake during dark hours.

Uptake of grass phytosiderophores by P. fluorescens in lieu of pyoverdine production may constitute an evolved strategy to thrive under Fe-deficient rhizosphere conditions. Metal deficiency has been shown to increase the number of fluorescent pseudomonads in the rhizosphere of wheat (52), suggesting some adaptation that enables them to outcompete other taxa. There is evidence that such changes in rhizosphere community composition may be largely controlled by plant exudates. Foliar application of Fe to barley grown in alkaline soils results in different rhizosphere communities with different spatial distributions, likely through decreased phytosiderophore secretion (53). The ability to sense and utilize host-derived phytosiderophores rather than producing endogenous siderophores could confer a competitive advantage over other microbes that invest resources into producing siderophores that compete with phytosiderophores for Fe binding.

Colonization by SBW25 had several effects on host metabolism that are relevant to plant health. Bacterial degradation of phytosiderophores appeared to result in upregulation of phytosiderophore biosynthesis, which likely had an energetic and nutrient cost to the host plant (54). However, SBW25 did not significantly affect overall plant growth in this study, and it is possible that secretion of phytosiderophores is one mechanism by which graminaceous plants encourage the growth of beneficial rhizosphere bacteria that can consume them in soils. Further work is needed to directly evaluate whether P. fluorescens assimilates C, N, or Fe associated with phytosiderophores and to understand the costs and benefits that determine how phytosiderophore-pirating bacteria affect plant health. Common mechanisms by which P. fluorescens promotes host health include competition against pathogens for carbon substrates, production of antibiotics, and induction of systemic resistance (55–57). Our study shows that SBW25 efficiently degrades the major sugars, small metabolites, and organic acids secreted by *B. distachyon*, which may reduce organic carbon and nutrient substrates available to pathogens in natural soils. SBW25 also induced transcription of genes likely involved in plant defense responses, including toxic cyanogenic glycoside production and oxidative stress management, while repressing others, such as antimicrobial thionin production. Similar effects of SBW25 inoculation were generally observed under Fe-replete and Fe-deficient growth conditions. Differential gene expression of regulatory DNA binding and plant hormone pathways (gibberellin and jasmonic acid) provides gene targets for future studies of the regulatory pathways that govern *B. distachyon* interactions with the root microbiome. Under the growth conditions of this study, P. fluorescens did not produce pyoverdines, which were previously shown to provide host protection against fungal pathogens, raising questions of whether pyoverdine production is repressed within the rhizosphere of graminaceous plants. The outcomes of this study provide insight into the molecular mechanisms by which P. fluorescens interacts with a graminaceous host under Fe-deficient conditions and provides targets for functional analyses that help evaluate the effect of root-associated bacteria on plant health.

## MATERIALS AND METHODS

### Materials and reagents.

Ultrapure 18.2-MΩ·cm water (qH_2_O) and mass spectrometry-grade organic solvents were used throughout the study. Plant growth containers consisted of lidded, transparent polycarbonate vessels (Magenta GA-7) with 140 g of 3-mm soda lime silica beads. Containers and beads were soaked in 10% trace metal grade HCl (Fisher Scientific) for 3 days to remove metal contamination and rinsed five times with qH_2_O. Containers were autoclaved to sterilize before use. Hoagland solution (1× strength) consisted of final concentrations of 1 mmol liter^−1^ NH_4_H_2_PO_4_, 6 mmol liter^−1^ KNO_3_, 4 mmol liter^−1^ Ca(NO_3_)_2_, 2 mmol liter^−1^ MgSO_4_, 0.46 μmol liter^−1^ H_3_BO_3_, 9.1 μmol liter^−1^ MnCl_2_, 0.77 μmol liter^−1^ ZnSO_4_, 0.50 μmol liter^−1^ CuSO_4_, and 0.11 μmol liter^−1^ H_2_MoO_4_, and 1 ml/liter of freshly prepared 0.1 mol liter^−1^ ferric EDTA stock. The 1× solution was diluted to 1/4 strength with qH_2_O and filter sterilized (0.2-μm polyethersulfone membrane) for all plant watering. For Fe-deficient treatments, Fe-EDTA was not added. The background 1/4-strength Hoagland solution Fe concentration prior to Fe amendment was below 0.1 ppb, as measured by inductively coupled plasma mass spectrometry. Bacterial cultures used to inoculate plants were grown in precultures of M9 minimal medium (1× M9 minimal salts with 0.2 mM CaCl_2_, 1 mM MgSO_4_, and 0.4% glucose).

### Plant and bacterial growth and physiology.

*B. distachyon* seeds from inbred line BD21 were sterilized by immersion in 70% ethanol for 30 s followed by immersion in a 1.3% bleach solution for 4 min and then rinsed for 30 s with sterile qH_2_O three times. All handling was conducted in a sterile laminar flow hood to avoid contamination. The sterile seeds were germinated on 1× Hoagland solution agar plates (25) in the dark at 4°C for 2 days followed by transfer of a total of 80 seedlings into 20 sterile growth containers (4 seedlings per container) that were placed in light boxes operating at 14:10 sunlight at 50% relative humidity and 20°C. After planting, 10 ml of 1/4-strength sterile Hoagland solution was added to each container, and the containers were covered with transparent lids and returned to the light boxes. The plants were watered every 3 to 4 days by removal of the medium via aspiration through a sterile pipette and addition of fresh Hoagland solution (10 ml). No significant evaporation of nutrient solution was observed between watering time points.

After establishing growth under the same conditions, each container received one of four treatments (total of 20 replicate plants per treatment): (i) no change in watering conditions (+Fe sterile), (ii) Fe deprivation (−Fe sterile), (iii) inoculation with SBW25, and (iv) inoculation with SBW25 and Fe deprivation. Plants that were inoculated with SBW25 were watered once with bacterium-containing Hoagland solution on day 14 of growth. For this, we used P. fluorescens strain SBW25::gfp/lux, which is chromosomally tagged with the *gfp-luxAB* gene cassette (58) to express green fluorescent protein (GFP) and bacterial luciferase to facilitate microscopic imaging. An individual colony of SBW25 on an M9 agar plate was inoculated into 10 ml of M9 medium and incubated at 30°C for 24 h. The numbers of bacteria were 7 × 10^6^ to 1 × 10^7^ cells/ml, based on plating out serial dilutions in replicate and counting discrete colonies. The culture was centrifuged at 4,500 rpm for 5 min, the supernatant was removed, and the pellet was resuspended in 200 ml of Hoagland solution. A 10-ml portion of each solution was added to plants receiving the bacterial inoculation treatment. For −Fe treatments, Fe was omitted from the Hoagland watering solution beginning on day 16 of growth for the remainder of the experiment.

Samples were harvested 28 days after planting in the growth containers at noon (after 7 h of light exposure). The nutrient solution in the container was transferred to a 15-ml polypropylene centrifuge tube, of which 100 μl was used for bacterial counts. The sterility of uninoculated plants was confirmed by plating nutrient solution onto LB agar medium and observing no colony formation. Bacterial counts were greater than 10^7^ CFU ml^−1^ in the growth solution of inoculated plants at the end of the experiment. The remainder of the nutrient solution was centrifuged at 4,500 rpm for 5 min at 4°C, and the bacterial pellet was processed for transcriptomic analysis. The supernatant was stored at −20°C for metabolomic analysis by LC-MS and NMR. One plant from each container was set aside for microscopy. Two plants were cut between the roots and shoots, and the sections were weighed on an analytical balance before the roots were immediately frozen in natural craft bags in liquid nitrogen and stored at −80°C prior to RNA extraction for transcriptomic analyses. Plants from the same container were pooled to obtain 5 biological replicates for each condition.

### Liquid chromatography-mass spectrometry.

A 1.6-ml volume of each sample was transferred into autosampler vials, dried by vacuum centrifugation, and dissolved in 100 μl of qH_2_O by sonicating for 5 min. The samples were centrifuged at 10,000 rpm for 5 min, and the supernatant was transferred to new vials with 300-μl-volume inserts for analysis. These samples were analyzed by liquid chromatography-mass spectrometry on a quadrupole time of flight (qTOF) mass spectrometer (Agilent Technologies). A 20-μl sample was injected on a hydrophilic end-capped C_18_ column (Thermo Hypersil gold AQ; 3-μm particle size, 2.1 by 100 mm with guard column). The compounds were separated at a 200-μl/min flow rate starting at 98% qH_2_O with 0.1% formic acid (solvent A):2% acetonitrile with 0.1% formic (solvent B) and holding for 10 min, followed by a 20-minute gradient to 97% solvent B over 20 min, and then isocratic flow at 97% B for 10 min. Compounds were detected in positive mode over a mass range of 100 to 1,500 *m/z*. Collision-induced fragmentation spectra were collected at 30 eV for major compounds. Quality control standards were analyzed before and after the sample sequence to ensure that chromatography and mass spectrometry performance was maintained. The LC-MS data were converted to open-source MzML file formats using MsConvert (proteowizard) (59), and pairwise comparative metabolomic analyses were conducted with XCMS using default qTOF parameters (60). Adducts and isotopologues were removed, and the masses of features exhibiting statistically significant differences between treatments were searched against the BioCyc and PlantCyc metabolic pathway databases to generate putative annotations based on matches to known molecules within predicted metabolic pathways of *B. distachyon* and P. fluorescens SBW25 (38, 61, 62).

### Confocal microscopy.

Sections of the *B. distachyon* roots were transferred to a sterile glass-bottom dish and immersed in Hoagland solution. The roots were imaged with an inverted confocal fluorescence microscope (Zeiss LSM 710) fitted with a 40×, 1.1 numerical aperture (NA), long-working-distance water immersion objective. The fluorescence excitation wavelength was 488 nm to excite the GFP of the bacteria, and the detection wavelength range was 492 nm to 563 nm. Fluorescence and transmitted light signals were recorded simultaneously. Three-dimensional (3D) confocal Z stacks were acquired to image the roots in depth.

### Nuclear magnetic resonance metabolomics.

Root exudate samples for NMR were concentrated 10-fold by vacuum centrifugation and diluted by 10% (vol/vol) with 5 mM 2,2-dimethyl-2-silapentane-5-sulfonate-d6 (DSS) as an internal standard. Metabolite concentrations were quantified by ^1^H NMR analysis. The one-dimensional (1D) ^1^H NMR spectra of all samples were collected in accordance with standard Chenomx (Edmonton, Alberta, Canada) sample preparation and data collection guidelines (63). NMR data for 5 replicate samples for each condition were acquired on a Varian Direct Drive (VNMRS) 600-MHz spectrometer (Agilent Technologies) equipped with a triple-resonance salt-tolerant cold probe with a cold carbon preamplifier. Varian standard 1D proton nuclear Overhauser effect spectroscopy (NOESY) with presaturation (TNNOESY) was carried out at 25°C with a nonselective 90° excitation pulse, a 100-ms mixing time, an acquisition time of 4 s, and a spectral width of 12 ppm. A presaturation delay of 1.5 s was used to optimize water suppression. The 1D ^1^H NMR spectra of all samples were processed, assigned, and analyzed by using Chenomx NMR Suite 8.3 with quantification based on spectral intensities relative to the internal standard using a previously described protocol (64). The detection limit of the method was ∼1 μM. Candidate metabolites present in each of the complex mixture were identified by matching the chemical shift, J coupling, and intensity information of experimental NMR signals against the NMR signals of standard metabolites in the Chenomx library.

### Transcriptomics.

RNA from *Brachypodium* roots was extracted using Qiagen Mini RNeasy kits, followed by genomic DNA removal using a Qiagen RNase-free DNase set kit. Integrity of the RNA samples was assessed using an Agilent 2100 Bioanalyzer. TruSeq stranded mRNA was used to generate cDNA libraries for sequencing (Illumina NextSeq550 sequencer). RNA from the bacterial pellet was extracted using TRIzol followed by cleanup using Qiagen Mini RNeasy kits and Qiagen RNase-free DNase sets. The SMARTerR Universal Low RNA kit was used to generate a full-length cDNA template library according to the instruction manual, follow by use of the Ion Plus fragment library kit (Thermo Fisher Scientific) to fragment cDNA and barcode adapter ligation for template preparation. The template library was quantified using the Ion Library TaqMan quantitation kit (Thermo Fisher Scientific). Emulsion clonal bead amplification was used to generate bead templates for the Ion Torrent platform using the Ion PI Hi-Q Chef kit with Ion PI Chip kit v3 on the Ion Chef system (Thermo Fisher Scientific). Samples were sequenced using the Ion PI Hi-Q Sequencing 200 kit (Thermo Fisher Scientific) on an Ion Proton sequencer with data processing through Torrent Suite software (v. 4.0.2). Read quality for both platforms was assessed and verified using fastqc (65). For Ion Proton data from SBW25, reads were aligned to genome GCA_000009225.1 using TMAP (available from https://github.com/iontorrent/TMAP), the aligner designed for Ion platform reads. For Illumina NextSeq reads from *Brachypodium distachyon*, reads were aligned to the genome Bdistachyon_314_v3.0 using Bowtie2 (66). Aligned reads were mapped to genes using htseq-count (67). Differential expression was performed with the R package DESeq2 (68). Full results from this analysis are provided in Data Set S1 and Data Set S2 in the supplemental material.

### Data availability.

Transcriptome sequencing (RNA-seq) data have been deposited in the National Center for Biotechnology Gene Expression Omnibus data repository under accession number GSE158273. Metabolomic data are available as a Mass Spectrometry Interactive Virtual Environment (MassIVE) data set (accession number MSV000086142).

10.1128/mSystems.00580-20.3 DATA SET S1 DESeq2 results, *B. distachyon*. Download Data Set S1, XLSX file, 10.2 MB. Copyright © 2021 Boiteau et al. 2021 Boiteau et al. This content is distributed under the terms of the Creative Commons Attribution 4.0 International license.

10.1128/mSystems.00580-20.4 DATA SET S2 DESeq2 results, P. fluorescens SBW25. Download Data Set S2, XLSX file, 0.6 MB. Copyright © 2021 Boiteau et al. 2021 Boiteau et al. This content is distributed under the terms of the Creative Commons Attribution 4.0 International license.
