# Supplementary material for: Metabolic Interactions between Brachypodium and Pseudomonas fluorescens under Controlled Iron-Limited Conditions
Source: mSystems. 2021 Jan 5;6(1):e00580-20. doi: 10.1128/mSystems.00580-20 (PMC7786132; doi:10.1128/mSystems.00580-20)
Supplement: TABLE S1 [file mSystems.00580-20-st001.pdf]

Table S1: Transcriptional response and adjusted P-value (Padj) of B. distachyon roots to SBW25 under +Fe and -Fe growth conditions including GO terms and pathways.

Gene ID	Log2 Fold Change*	Std Error	Adjusted P-value	Log2 Fold Change*	Std error	Adjusted P-value	Annotation	GO Term~	Pathway#
	+Fe treatments			-Fe treatments					
**Biosynthesis and metabolism**									
Bradi2g11870	**-1.12**	**0.28**	**7.4E-03**	**-0.89**	**0.28**	**4.9E-02**	glycosyl hydrolase	Catalytic activity (GO:0003824)	cellulose biosynthesis (PWY-1001)
Bradi1g00950	**-1.06**	**0.26**	**5.0E-03**	-0.58	0.25	1.4E-01	2-oxoglutarate oxygenase	Oxidoreductase activity (GO:0016491)	gibberellin biosynthesis (PWY-5035)
Bradi2g49067	**1.01**	**0.28**	**1.5E-02**	0.72	0.28	9.5E-02	Uridine-diphospho (UDP) - glucosyl transferase	Metabolic process (GO:0008152)	apigeninidin 5-O-glucoside biosynthesis (PWY-7253)
Bradi1g22340	**1.01**	**0.28**	**1.5E-02**	0.13	0.27	8.1E-01	cytochrome P450	Oxidoreductase activity (GO:0016705)	dhurrin biosynthesis (PWY-861)
Bradi4g31960	**1.03**	**0.21**	**6.1E-04**	0.53	0.21	1.1E-01	Pyridoxal phosphate-dependent transferase		L-methionine salvage cycle II (PWY-7270)
Bradi4g43610	**1.04**	**0.28**	**1.4E-02**	0.47	0.28	2.9E-01	2'-deoxymugineic-acid 2'-dioxygenase	Oxidoreductase activity (GO:0016491)	phytosiderophore biosynthesis (PWY-5925)
Bradi3g13700	**1.05**	**0.24**	**2.1E-03**	0.56	0.23	1.2E-01	D-arabinono-1,4-lactone oxidase	Catalytic activity (GO:0003824)	
Bradi3g18160	**1.05**	**0.28**	**1.4E-02**	0.77	0.28	8.1E-02	D-arabinono-1,4-lactone oxidase	Catalytic activity (GO:0003824)	
Bradi4g08830	**1.05**	**0.27**	**8.9E-03**	**0.87**	**0.27**	**4.4E-02**	Aldolase-type triose phosphate isomerase (TIM) barrel	Metabolic process (GO:0008152)	L-tryptophan biosynthesis (TRPSYN-PWY)
Bradi3g06330	**1.05**	**0.26**	**6.5E-03**	**1.14**	**0.26**	**4.1E-03**	cytochrome P450	Oxidoreductase activity (GO:0016705)	
Bradi1g59570	**1.05**	**0.28**	**1.2E-02**	-0.57	0.28	1.8E-01	gibberellin 2-oxidase	Oxidoreductase activity (GO:0016491)	
Bradi4g43380	**1.06**	**0.22**	**4.1E-04**	0.55	0.21	1.0E-01	cytochrome P450	Oxidoreductase activity (GO:0016705)	jasmonoyl-L-isoleucine inactivation (PWY-7859)
Bradi1g06730	**1.12**	**0.27**	**5.1E-03**	0.81	0.27	6.1E-02	cytochrome P450	Oxidoreductase activity (GO:0016705)	
Bradi2g44150	**1.14**	**0.24**	**7.0E-04**	0.68	0.24	7.2E-02	cytochrome P450	Oxidoreductase activity (GO:0016705)	
Bradi2g44160	**1.28**	**0.27**	**6.8E-04**	**0.89**	**0.27**	**4.0E-02**	cytochrome P450	Oxidoreductase activity (GO:0016705)	
Bradi1g64120	**1.32**	**0.24**	**2.9E-05**	-0.10	0.23	8.2E-01	galactinol synthase		stachyose biosynthesis (PWY-5337)
**Stress and Defense**									
Bradi1g57280	**-1.26**	**0.28**	**2.1E-03**	**-1.24**	**0.28**	**4.4E-03**	Plant thionin	Defense response (GO:0006952)	
Bradi1g57285	**-1.17**	**0.25**	**7.1E-04**	**-1.17**	**0.24**	**1.5E-03**	Plant thionin	Defense response (GO:0006952)	

Bradi4g14920	**0.85**	**0.27**	**3.9E-02**	**1.01**	**0.27**	**1.8E-02**	Chitinase	Defense response (GO:0042742)
Bradi5g27170	**-1.36**	**0.27**	**2.6E-04**	-0.82	0.26	5.1E-02	Peroxidase	Response to oxidative stress (GO:0006979)
Bradi2g20850	**-1.35**	**0.27**	**2.8E-04**	**-1.05**	**0.27**	**1.2E-02**	Peroxidase	Response to oxidative stress (GO:0006979)
Bradi1g38310	**-1.12**	**0.28**	**6.1E-03**	-0.80	0.27	6.6E-02	Peroxidase	Response to oxidative stress (GO:0006979)
Bradi4g24650	-0.57	0.23	1.2E-01	**-1.06**	**0.23**	**2.2E-03**	Abscisic acid or water deficit stress (ABA/WDS) induced protein	Response to stress (GO:0006950)
Bradi2g37060	0.21	0.26	6.7E-01	**1.32**	**0.26**	**6.6E-04**	Peroxidase	Response to oxidative stress (GO:0006979)
Bradi3g55850	**1.18**	**0.27**	**2.8E-03**	0.42	0.27	3.1E-01	Peroxidase	Response to oxidative stress (GO:0006979)
Bradi4g25660	**1.20**	**0.28**	**4.5E-03**	0.60	0.28	1.7E-01	Peroxidase	Response to oxidative stress (GO:0006979)
**Transport**								
Bradi5g14400	0.57	0.20	6.7E-02	**1.00**	**0.20**	**6.8E-04**	Oligopeptide transport related	Transport (GO:0006810)
Bradi5g14410	0.65	0.25	9.7E-02	**1.03**	**0.25**	**7.8E-03**	Oligopeptide transmembrane transporter	Transport (GO:0006810)
Bradi3g35390	**1.01**	**0.28**	**1.9E-02**	0.57	0.28	2.0E-01	Adenylpyrophosphatase (ATPase) activity	Transport (GO:0006810)
**Regulation**								
Bradi1g56841	0.45	0.25	2.6E-01	**1.11**	**0.24**	**2.3E-03**	FAR1 DNA-binding domain containing protein	Regulation of transcription (GO:0006355)
Bradi1g58057	**1.00**	**0.24**	**5.4E-03**	**1.04**	**0.24**	**4.7E-03**	NAC domain containing protein	Regulation of transcription (GO:0006355)
Bradi3g22040	**1.07**	**0.23**	**1.0E-03**	0.17	0.23	6.6E-01	Basic leucine zipper (bZIP) transcription factor	Regulation of transcription (GO:0006355)
Bradi2g40582	**1.28**	**0.25**	**2.8E-04**	0.20	0.25	6.5E-01	bZIP transcription factor	Regulation of transcription (GO:0006355)
Bradi3g07540	**1.74**	**0.27**	**3.4E-07**	-0.27	0.26	5.3E-01	bZIP transcription factor	Regulation of transcription (GO:0006355)

*+SBW25/-SBW25 ± standard error
~GO terms obtained from PhytoMine (https://phytozome.jgi.doe.gov/phytomine)
Pathways obtained from PlantCyc (https://pmn.plantcyc.org)
