# Supplementary material for: Metabolic Interactions between Brachypodium and Pseudomonas fluorescens under Controlled Iron-Limited Conditions
Source: mSystems. 2021 Jan 5;6(1):e00580-20. doi: 10.1128/mSystems.00580-20 (PMC7786132; doi:10.1128/mSystems.00580-20)
Supplement: TABLE S2 [file mSystems.00580-20-st002.pdf]

Table S2: Transcriptional response of P. fluorescens SBW25 to Fe deprivation: genes with predicted pyoverdine biosynthesis functions.*

Gene ID	Gene*	Log2 Fold Change**	Std Error	Adjusted P-value	Description
gene-PFLU_RS10025		0.01	0.30	1.00	metal ABC transporter substrate-binding protein
gene-PFLU_RS10030		-0.39	0.32		metal ABC transporter permease
gene-PFLU_RS10035		0.01	0.33	1.00	metal ABC transporter ATP-binding protein
gene-PFLU_RS10040		0.10	0.33	0.98	ABC transporter substrate-binding protein
gene-PFLU_RS10045		-0.37	0.32	0.89	hypothetical protein
gene-PFLU_RS10050		0.37	0.29		hypothetical protein
gene-PFLU_RS10055		-0.19	0.33		hypothetical protein
gene-PFLU_RS10060		-0.19	0.29	0.95	PepSY domain-containing protein
gene-PFLU_RS12435	pvdI	-0.14	0.24	0.95	non-ribosomal peptide synthetase
gene-PFLU_RS12440		0.06	0.18	0.98	non-ribosomal peptide synthetase
gene-PFLU_RS12445	fpvA	-0.19	0.23	0.94	TonB-dependent siderophore receptor
gene-PFLU_RS12450	pvdE	-0.20	0.31	0.95	cyclic peptide export ABC transporter
gene-PFLU_RS12455	pvdF	-0.24	0.31	0.94	N(5)-hydroxyornithine transformylase PvdF
gene-PFLU_RS12460	pvdO	-0.47	0.33	0.87	formylglycine-generating enzyme family protein
gene-PFLU_RS12465	pvdN	0.20	0.31	0.95	aminotransferase class V-fold PLP-dependent enzyme
gene-PFLU_RS12470	pvdM	-0.24	0.29	0.94	membrane dipeptidase
gene-PFLU_RS12475	pvdP	0.40	0.26	0.85	hypothetical protein
gene-PFLU_RS12480		0.21	0.21	0.92	non-ribosomal peptide synthetase
gene-PFLU_RS12485		0.21	0.22	0.93	non-ribosomal peptide synthetase
gene-PFLU_RS13190	fpvR	-0.37	0.24	0.85	FecR family protein
gene-PFLU_RS16355	pvdQ	-0.27	0.28	0.93	acylase
gene-PFLU_RS19415	pvdA	-0.29	0.31	0.93	L-ornithine 5-monooxygenase
gene-PFLU_RS19420	fpvI	-0.22	0.33	0.95	RNA polymerase sigma factor
gene-PFLU_RS19425		-0.45	0.28	0.83	efflux RND transporter periplasmic adaptor subunit
gene-PFLU_RS19430		-0.36	0.26	0.87	MacB family efflux pump subunit
gene-PFLU_RS19435	opmQ	0.14	0.32	0.97	RND transporter
gene-PFLU_RS21465		-0.55	0.33	0.81	MbtH family protein
gene-PFLU_RS21470	pvdH	-0.03	0.32	0.99	diaminobutyrate--2-oxoglutarate transaminase
gene-PFLU_RS21515	pvdL	-0.10	0.24	0.97	non-ribosomal peptide synthetase
gene-PFLU_RS21520	pvdG	0.22	0.33	0.95	thioesterase
gene-PFLU_RS21525	pvdS	0.15	0.31	0.97	RNA polymerase factor sigma-70

*Predicted genes as described by Moon et al., 2008. ; Moon, C.D., Zhang, X.X., Matthijs, S., Schäfer, M., Budzikiewicz, H., Rainey, P.B., 2008. Genomic, genetic and structural analysis of pyoverdine-mediated iron acquisition in the plant growth-promoting bacterium Pseudomonas fluorescens SBW25. BMC Microbiol. 8, 1–13.

**-Fe/+Fe treatments
